# Endocrine-metabolic modulators in sperm biotechnology: cross-species mechanisms and translational priorities

**DOI:** 10.3389/fvets.2026.1920393

**Published:** 2026-09-21

**Authors:** Pallav Sengupta, Sulagna Dutta, Sanaz Alaee, Deepali Mishra, Manesh Kumar Panner Selvam, Israel Maldonado Rosas, Shubhadeep Roychoudhury

**Affiliations:** 1 Department of Biomedical Sciences, College of Medicine, Gulf Medical University, Ajman, United Arab Emirates; 2 Basic Medical Sciences Department, College of Medicine, Ajman University, Ajman, United Arab Emirates; 3 Center of Medical and Bio-Allied Health Sciences Research, Ajman University, Ajman, United Arab Emirates; 4 Department of Reproductive Biology, Shiraz University of Medical Sciences, Shiraz, Iran; 5 Center for Research in Pure and Applied Science, Jain Deemed-to-be University, Bengaluru, India; 6 Department of Urology, Tulane University School of Medicine, New Orleans, LA, United States; 7 Citmer Reproductive Medicine, Mexico City, Mexico; 8 Department of Life Science and Bioinformatics, Assam University, Silchar, India

**Keywords:** adipokines, fertility biomarkers, insulin resistance, sperm cryopreservation, vitamin D

## Abstract

Endocrine-metabolic dysregulation is increasingly recognized as a determinant of male reproductive competence. Its implications for sperm biotechnology remain under-integrated. This review advances a systems framework in which insulin resistance (IR), adipokine imbalance, and vitamin D-calcium signaling converge on core sperm functional modules, i.e., membrane lipid architecture, mitochondrial efficiency, redox stability, and capacitation kinetics. Across species, metabolic strain is associated with oxidative vulnerability and altered substrate handling – amplifying damage during cooling, cryopreservation, and sorting. Adipokines emerge as biomarkers of energy-inflammatory state and as functional modulators within seminal plasma and at the sperm surface, with dose- and context-dependent effects on motility and survival. The vitamin D axis, through Ca^2+^-gated signaling, intersects with motility architecture and activation timing, suggesting compatibility constraints during selection and post-thaw use. By mapping endocrine inputs onto specific biotechnology “pinch-points”, we propose an endotype-driven model for donor stratification and protocol customization. Stress-response phenotyping, function-rich endpoints beyond conventional motility, and standardized reporting of processing metadata are emphasized to enable cross-study comparability. Stratified trials, process-aware outcome measures, and reverse translation of functional assays between animal and human contexts are translational priorities. Current evidence supports further investigation of donor-stratified metabolic phenotyping as a potential approach to sperm processing, with the aim of reducing sublethal dysfunction and improving predictability of fertility under real-world assisted reproduction workflows. In veterinary reproduction, this framework is especially relevant to artificial insemination-center donor stratification, cryobank intake, chilled-semen transport, and quality control of frozen or sex-sorted semen doses.

## 1 Introduction

The decline in reproductive efficiency in livestock and companion animals is increasingly viewed through a metabolic lens. Beyond the established roles of spermatogenesis and epididymal maturation, systemic endocrine-metabolic disturbances, including insulin resistance (IR), adipokine imbalance, and hypovitaminosis D, have been associated with altered semen quality, oxidative vulnerability, and impaired fertilizing capacity across species (1, 2). In livestock, adverse energy balance, excessive adiposity, and subclinical inflammatory states are now recognized as modifiers of testicular steroidogenesis and epididymal function, with measurable consequences for sperm membrane integrity, mitochondrial efficiency, and capacitation competence (3). These effects are particularly relevant to sperm biotechnology because the metabolic condition of the donor may influence how sperm respond to collection, cooling, freezing, thawing, sorting, and selection.

Cryopreservation, sex-sorting, and selection platforms depend on the intrinsic biophysical and metabolic characteristics of spermatozoa. However, sperm derived from metabolically compromised males show altered lipid architecture, enhanced reactive oxygen species (ROS) production, and impaired calcium signaling, characteristics that augment damage during cooling, osmotic stress, and flow cytometric maneuvering (4, 5). Adipokines such as leptin and adiponectin have been localized within the male reproductive tract and seminal plasma, where they seem to regulate motility and energy metabolism, indicating their candidacy as functional biomarkers rather than passive associates (6). Simultaneously, vitamin D signaling has been suspected in sperm calcium homeostasis and motility regulation in experimental as well as domestic species (7). Despite these observations, the available evidence remains fragmented. Studies often examine individual metabolic factors, animal species, or semen-processing procedures in isolation, making it difficult to determine which findings are biologically consistent, species-specific, or directly applicable to assisted breeding.

The rationale for this review is that endocrine-metabolic status may represent a measurable source of variation in semen processability, rather than only a background characteristic of the donor. The most immediate veterinary applications include AI-stud screening, semen-dose quality assurance, cryobank intake decisions, bull/stallion/boar donor management, and interpretation of unexplained between-donor variation in freezability (Table 1). Instead of treating metabolic dysregulation as an external confounder, it may be more productively considered a stratification axis influencing extender formulation, cryosurvival prediction, and fertility forecasting.

**Table 1:** Veterinary decision matrix for integrating endocrine-metabolic phenotyping into donor stratification, semen processing, and fertility prediction.

Veterinary setting	Metabolic/endocrine signal of concern	Minimum assessment set	Main sperm-biotechnology risk	Recommended processing or QC response	Preferred downstream endpoint	Species in which evidence is strongest	Key references
AI-stud donor intake	Overconditioning/suspected metabolic strain	Body condition; diet history; semen QC; oxidative marker if feasible	Hidden freezability defect despite acceptable baseline motility	Add stress-sensitive QC before routine inclusion	Post-thaw longevity; mitochondrial potential	Bull, boar	(19, 28, 30, 54, 55)
Cryobank intake	High seminal leptin/oxidative burden	Seminal leptin + MDA or equivalent oxidative marker	Reduced cryosurvival; shortened post-thaw lifespan	Flag donor as higher-risk; test tailored freezing protocol before banking	Post-thaw membrane integrity; acrosome status; longevity	Buffalo bull	(39, 40)
Chilled-semen transport	Adipokine-related motility instability	Seminal plasma profile where feasible; baseline motility kinetics	Rapid functional decline during storage	Use shorter storage window and intensified QC	Time-dependent motility and viability	Buffalo, pig	(11, 12, 39, 40)
Bull cryopreservation	IR-linked energetic dysregulation	Metabolic phenotype proxy + semen function panel	Mitochondrial dysfunction; acrosomal loss	Consider insulin-tested extender only under dose-validated conditions	Motility, plasma membrane function, acrosome integrity	Bull	(22, 23)
Boar freezing program	Excess substrate load during cooling	Medium composition review; donor-specific post-thaw history	Cryo-injury amplified by glycolytic overdrive	Lower glucose during pre-treatment/cooling	Post-thaw motility; fertilization-linked quality	Boar	(59)
Ram semen freezing	Vitamin D–Ca^2+^ timing sensitivity	Vitamin D status or seasonal proxy; semen function panel	Premature activation or poor post-thaw motility	Test vitamin D-enriched extender in controlled protocol	Post-thaw motility and viability	Ram	(52, 54)
Bull semen freezing	Variable vitamin D responsiveness	Baseline semen phenotype + vitamin D stratification	Inconsistent viability response after thaw	Restrict vitamin D extender use to phenotype-stratified testing	Viability; apoptosis-related indices	Bull	(53)
Poultry breeding / AI	Elevated chemerin / inflammatory reproductive state	Seminal chemerin if feasible; motility; sperm concentration	Reduced insemination fertility despite acceptable semen appearance	Prioritize donor ranking and exclusion thresholds	Fertility after AI; motility persistence	Rooster/chicken	(38)
Sex-sorting program	Oxidative/metabolic fragility	Oxidative status; mitochondrial function; donor freezability history	Higher ROS burden; DNA and mitochondrial stress after sorting	Pre-sort stratification and intensified post-sort recovery QC	Post-sort viability; ROS; DNA integrity	Bull, stallion	(5, 58, 65–69)
Sperm-selection workflow	Timing-sensitive Ca^2+^/motility phenotype	Vitamin D axis proxy + motility architecture	Selection of fast but short-lived sperm subpopulations	Match method to donor phenotype; emphasize post-selection durability	Post-selection longevity; IVF/fertilization outcome	Bull, boar, stallion	(70–74)
Fertility prediction model	Multidomain endocrine–metabolic burden	Conventional semen + oxidative marker + selected adipokine + process metadata	Poor prediction if only routine semen parameters are used	Use multivariate, process-aware prediction framework	Fertility calibration, post-thaw classification accuracy	Bull, multi-species	(75–79)

Accordingly, the main objective of this review is to evaluate how IR-associated metabolic strain, adipokine imbalance, and vitamin D-calcium signaling influence sperm functional resilience and biotechnology-related outcomes across animal species. Specifically, the review addresses four questions: whether these endocrine-metabolic factors alter sperm membrane, mitochondrial, redox, and capacitation-related functions; whether their effects become more apparent during cooling, cryopreservation, sex-sorting, or sperm selection; whether the available evidence supports donor stratification or protocol customization; and which methodological gaps must be addressed before these markers can be incorporated into routine veterinary practice. The rationale of this review is to determine whether endocrine-metabolic phenotyping can explain donor-to-donor variation in sperm function and processing resilience, and whether this information can support species-specific and procedure-specific semen management. The review therefore evaluates how IR, adipokine imbalance, and vitamin D-calcium signaling influence sperm functional characteristics and outcomes after cooling, cryopreservation, sex-sorting, and selection. It also examines their potential value for donor stratification, protocol customization, and fertility prediction. Evidence that remains indirect is interpreted as hypothesis-generating rather than clinically or operationally established, while veterinary studies reporting semen-processing or fertility outcomes are given priority over human-based inference.

To synthesize the present narrative review, a structured literature search was conducted in PubMed, Scopus, and Web of Science. Search terms were combined using Boolean operators and included IR, metabolic syndrome, adipokines, leptin, adiponectin, vitamin D, sperm, semen, cryopreservation, sex-sorting, sperm selection, post-thaw function, and relevant animal-species terms. Peer-reviewed original studies and relevant reviews examining endocrine-metabolic factors in relation to sperm function, semen processing, cryosurvival, donor stratification, or fertility outcomes were considered. Studies without direct relevance to male reproduction or sperm biotechnology were excluded. Reference lists of relevant articles were also screened for additional publications. Evidence was synthesized narratively. For interpretation, greatest translational weight was given to direct biotechnology intervention studies, followed by animal intervention or fertility-linked studies, mechanistic studies, and observational or supportive human evidence. Observational associations were not interpreted as evidence of causality.

## 2 Crosstalk of endocrine-metabolic signals and sperm biotechnology

### 2.1 Endocrine-metabolic state variables

Endocrine-metabolic conditions in breeding males are often inferred from a limited set of systemic intermediaries, yet these cues disseminate across the hypothalamic-pituitary-gonadal axis and peripheral tissues in ways that can substantially alter the sperm phenotype presented to biotechnological processes. IR is usually coupled with hyperinsulinemia, dyslipidemia, low-grade inflammation, and heightened oxidative tone. These features have been associated with compromised semen parameters and higher vulnerability to oxidative insults in spermatozoa, especially in human observational cohorts and metabolic disorder conditions (2, 8, 9). Notably, the mechanistic significance of sperm biotechnology is less about IR as a diagnostic marker and more about the downstream biochemical conditions it develops, excessive ROS, advanced glycation end-products, and dysregulated lipid handling, each of which can reduce membrane stability, mitochondrial competence, and chromatin integrity (3, 9, 10).

Adipokines serve as endocrine indicators of energy balance and inflammatory tone within the same system. In the seminal plasma of several species, they are increasingly detectable, with receptor expression noted in the male reproductive tract and sperm cells. In porcine spermatozoa and seminal plasma, leptin signaling has been reported, bolstering a probable autocrine/paracrine regulatory loop that can regulate sperm function (11, 12). A broader reproductive regulatory footprint for adiponectin has been outlined, spanning steroidogenesis and oxidative-stress regulation in male gonads. This lays out a mechanistic bridge between systemic metabolic status and gamete quality (13). Across domestic species, however, adipokine biology remains susceptible to assay non-equivalence, breed/season confounding, and “biomarker drift” with age and adiposity. This makes it difficult to directly interpret decision thresholds for semen processing.

The vitamin D axis adds a discrete layer, *i.e*. it is not only a nutritional biomarker but also a regulator of calcium homeostasis. This axis is firmly associated with sperm motility and capacitation signaling. Human evidence supports connections between vitamin D status and sperm motility. Mechanistic studies have emphasized calcium-dependent pathways as probable mediators (7, 14). The impacts of supplementation, however, remain heterogeneous so far, indicating that vitamin D should be conceived not as a universal modifier but instead as a stratifying factor whose biological impact depends on its interaction with the underlying endocrine-metabolic and redox milieu. Thus, these variables define a practical phenotyping triad, IR-associated redox-lipid strain, adipokine signaling balance, and vitamin D-calcium homeostasis, whose combined state is likely to influence how sperm tolerate processing, sorting, and storage (Figure 1).

**Figure 1:**
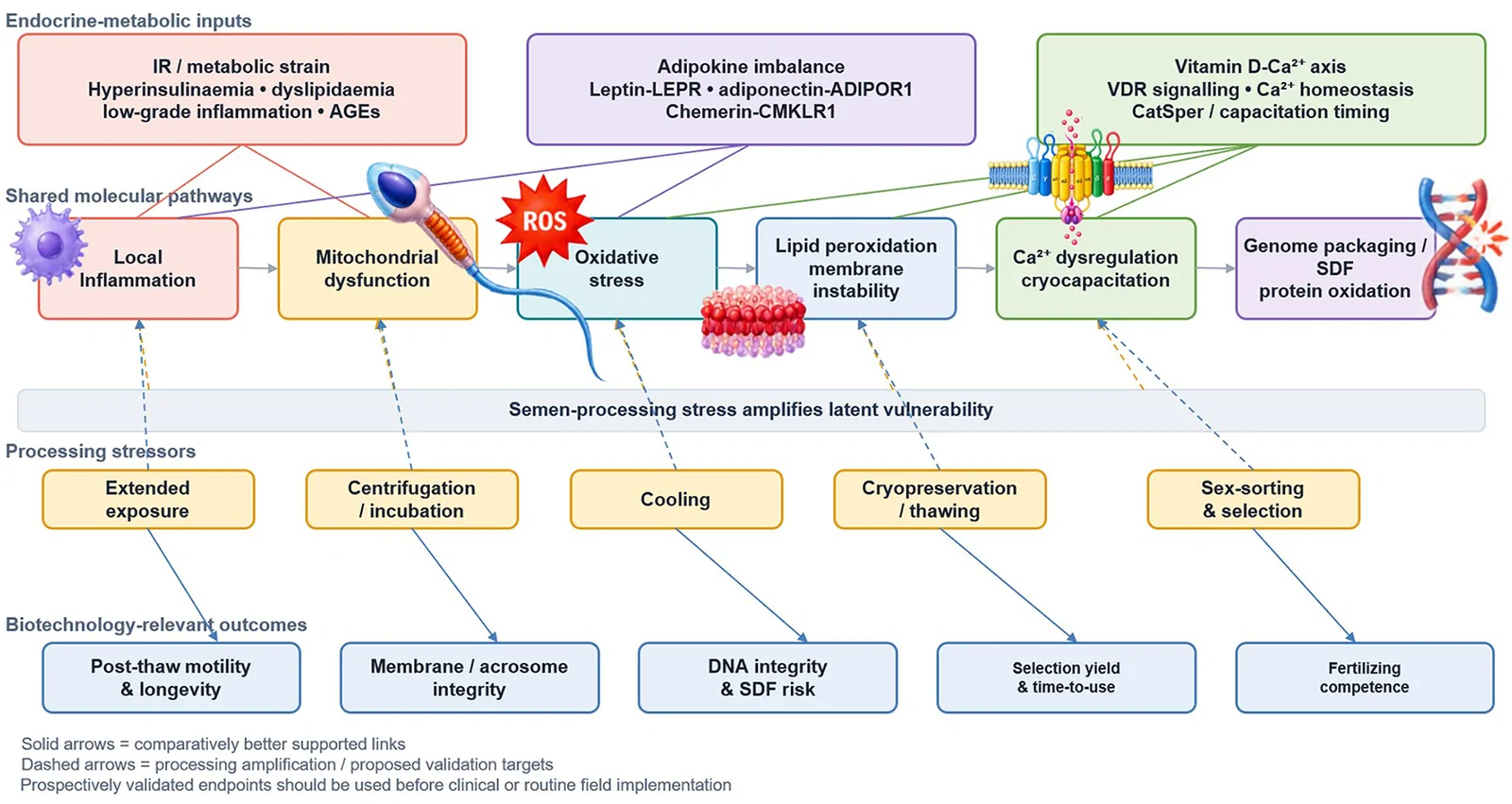
Proposed mechanistic framework linking endocrine-metabolic dysregulation with sperm functional resilience and biotechnology outcomes. Insulin resistance (IR) and associated metabolic strain promote low-grade inflammation, mitochondrial dysfunction, oxidative stress, and altered lipid handling, thereby increasing membrane instability and susceptibility to sperm DNA fragmentation. Adipokine imbalance, including altered leptin-LEPR, adiponectin-ADIPOR1, and chemerin-CMKLR1 signaling, further modifies inflammatory, metabolic, mitochondrial, and redox pathways that regulate sperm survival and function. In parallel, vitamin D-VDR signaling influences Ca^2+^ homeostasis and CatSper-mediated calcium entry, thereby affecting motility, capacitation timing, and the risk of cryocapacitation. These pathways converge on mitochondrial efficiency, ROS generation, lipid peroxidation, membrane and acrosomal integrity, Ca^2+^ signaling, genome stability, and ultimately sperm motility, longevity, DNA integrity, selection efficiency, and fertilizing competence. Semen-processing procedures, including prolonged ex vivo exposure, centrifugation, incubation, cooling, cryopreservation/thawing, sex-sorting, and sperm selection, may amplify these pre-existing vulnerabilities.

### 2.2 Sperm functional modules: structure-to-function mapping

Sperm biotechnology predominantly manipulates a cell that is simultaneously streamlined and fragile, *i.e*. minimal cytoplasm, high membrane specialization, and heavy reliance on compartmentalized signaling. For conceptual clarity, endocrine-metabolic modulators can be mapped onto four interdependent functional modules: (a) membrane lipid architecture, (b) mitochondrial efficiency and redox leakage, (c) capacitation-calcium signaling, and (d) genome packaging integrity. This mapping matters because biotechnology endpoints, post-thaw motility, acrosome integrity, sorting yield, and fertilization competence, are emergent outputs of these modules rather than isolated traits.

Membrane lipid architecture is a key deciding factor of cold-shock tolerance and osmotic resilience. Species differences in cholesterol: phospholipid ratios are familiar to shape cryosurvival, and targeted cholesterol loading has been exploited to improve resistance to cryoinjury (15). Endocrine-metabolic stressors that alter lipid handling and promote lipid peroxidation are therefore positioned to shift membrane phase behavior and destabilize the very parameter that extenders and cryoprotectants attempt to preserve (3, 10). This relationship provides a mechanistic foundation for why metabolic phenotyping may predict “freezability” beyond routine semen parameters.

Mitochondrial competence represents a balance between ATP production and ROS leakage. Oxidative stress (OS) has been persistently implicated in impaired sperm motility, sperm membrane injury, and sperm DNA fragmentation (SDF), with sperm being particularly vulnerable due to limited antioxidant capacity and high content of polyunsaturated fatty acids (3, 16). During cryopreservation, ROS production and antioxidant depletion can be amplified, facilitating structural and functional reduction that persists after thawing (5, 17). Hence, systemic metabolic states that prime mitochondria toward inefficient electron flow may pre-load sperm for higher processing losses.

Capacitation-calcium signaling constitutes a third module that is easily perturbed by both metabolic dysregulation and processing-induced “cryocapacitation”. The redox environment participates in physiologic capacitation but becomes pathologic when ROS exceeds the signaling window, and cryopreservation can distort this balance (18). Vitamin D-calcium homeostasis intersects here: vitamin D has been shown to increase intracellular calcium in human sperm and is associated with motility, providing a plausible mechanism for differential responses across vitamin D strata (7, 14).

Finally, genome packaging integrity is not a silent passenger. OS can increase SDF and protein oxidation, compromising the genomic payload delivered to the oocyte and potentially influencing embryo competence (10, 16). In practice, these modules are coupled: membrane injury propagates calcium dysregulation; mitochondrial stress increases ROS; and ROS damages membranes and DNA. A module-based framing, therefore, provides a mechanistic bridge from endocrine-metabolic variables to biotechnology failure points and offers testable hypotheses for stratified processing protocols.

### 2.3 Biotechnology pinch-points: small biological shifts to large process losses

Sperm biotechnology workflows impose sequential stress, dilution, cooling, exposure to cryoprotectants, freezing-thawing, centrifugation, flow cytometric sorting, and selection by migration or charge, each acting as a non-physiological filter. “Pinch-points” are the steps where modest pre-existing biological differences amplify into disproportionate losses of viable, fertilization-competent sperm. Endocrine-metabolic perturbations are hypothesized to act as latent multipliers at these pinch-points by pre-shaping membrane order, mitochondrial set-point, and redox reserves before processing even begins.

Cooling and cryopreservation remain dominant pinch-points. Decreased fertility with cryopreserved semen has long been ascribed to membrane phase transitions, osmotic injury, and OS. These processes converge on motility decrease, acrosomal disruption, and shortened lifespan after thawing (5). Contemporary biochemical characterizations in livestock emphasize that cryopreservation is not a single insult but a cascade, cholesterol redistribution, ROS accumulation, mitochondrial depolarization, and signaling derangements that resemble premature capacitation (“cryocapacitation”) (17, 18). When systemic metabolic dysfunction increases baseline oxidative tone, the distance to the damage threshold narrows; post-thaw competence may then become highly sensitive to small deviations in freezing curves, cryoprotectant concentration, or handling time (3, 10).

A different kind of pinch-point is represented by sorting and selection through the imposition of mechanical shear, altered media, and time-dependent stress, while simultaneously enriching specific motility or surface-property subpopulations. Leptin signaling components in spermatozoa and seminal plasma have been found to support the possibility that endocrine cues modulate sperm motility and signal transduction, which, in turn, can directly influence selection bias and post-processing performance in boar and pig models (11, 12). If adipokine imbalances modulate energetic strategy or motility patterning, then migration-based selection may preferentially retain a metabolically defined subset, potentially decoupling ‘selected sperm quality' from the baseline semen quality and complicating fertility prediction of men.

Additional pinch-points include extender transitions and osmotic steps. One of the principal determinants of cold-shock resistance is membrane cholesterol content. Cholesterol loading strategies have improved cryosurvival across species, underscoring that membrane composition is both a vulnerability and a tunable target (15). This becomes directly relevant when the endocrine-metabolic state alters lipid handling or oxidative susceptibility.

Vitamin D-calcium signaling introduces a final pinch-point logic, *i.e*. calcium flux is central to motility and capacitation timing, and vitamin D has been linked to intracellular calcium changes in sperm, suggesting that vitamin D strata could influence ‘time-to-capacitation' during processing and after thaw (7, 14). This raises a critical operational implication, *i.e*. rather than assuming uniform process robustness, donors may differ in where their pinch-point lies, cooling sensitivity, oxidative collapse post-thaw, or selection-induced exhaustion, arguing for phenotyping-based stratification and step-specific quality controls.

## 3 Cross-species evidence map: IR and sperm biotechnology outcomes

IR and related endocrine-metabolic disturbances may influence sperm function and its response to biotechnology procedures through changes in energy metabolism, oxidative balance, membrane stability, and mitochondrial activity. Evidence from cattle, stallions, boars, experimental models, and human studies provides complementary insights into these relationships, ranging from field observations and mechanistic investigations to interventions applied directly during semen processing. The following species-based sections examine the relevance of these findings to cooling, cryopreservation, sperm selection, and donor stratification. Representative evidence is summarized in Table 2.

**Table 2:** Cross-species evidence linking insulin resistance (IR), adipokines, and vitamin D to sperm functional modules and biotechnology outcomes in veterinary reproduction.

Species	Modulator	Biological compartment studied	Principal sperm functional module affected	Biotechnology-relevant outcome	Direction of effect	Evidence level	Veterinary interpretation/application	Key reference
Bull/cattle	High-energy diet/metabolic strain	Whole animal	Redox balance; membrane stability; semen quality resilience	Impaired semen quality; possible embryo-development effects	Mostly adverse	Observational + integrative review	Supports metabolic screening in AI studs and cryobank donor intake	(20, 21)
Stallion	Adiposity without insulin dysregulation	Whole animal	Post-thaw semen traits	No clear association with post-thaw semen parameters	Neutral	Observational	Body condition alone is insufficient as a processing-risk marker	(24)
Bull	Insulin supplementation	Extender/cryopreservation system	Mitochondrial function; motility; acrosome stability	Improved post-thaw motility and membrane function at some doses; possible acrosomal compromise at higher dose	Biphasic	Direct biotech intervention	Insulin should be tested as a dose-calibrated extender additive, not used empirically	(22, 23)
Boar	Glucose reduction in pre-treatment medium	Cooling/cryopreservation step	Energy handling; motility; cryostress response	Improved post-thaw sperm quality	Beneficial	Direct biotech intervention	Substrate control is a practical lever in boar cryobiology	(59)
Pig/boar	Leptin/leptin receptor	Spermatozoa; seminal environment	Motility signaling; capacitation; survival	Functional involvement suggested	Context-dependent	Mechanistic	Supports receptor-informed stratification studies in semen processing	(11, 12)
Buffalo bull	Seminal leptin	Seminal plasma	Oxidative vulnerability; abnormal morphology	Higher leptin associated with abnormalities and MDA	Adverse	Observational biomarker study	Candidate biomarker for cryobank intake risk and post-processing vulnerability	(39)
Buffalo bull	Leptin added *in vitro*	Extender/cooled semen	Motility; viability	Low dose beneficial, higher dose detrimental	Biphasic	Direct biotech intervention	Classic example of narrow therapeutic window for adipokine-based modulation	(40)
Rooster/chicken	Chemerin	Seminal plasma; *in vitro* sperm exposure	Motility; fertility competence	Reduced sperm motility and fertility	Adverse	Mechanistic + fertility-linked	High-priority biomarker candidate in poultry breeding systems	(38)
Ram	ADIPOR1 axis	Testis and accessory glands	Tract-level adipokine responsiveness	Indirect relevance to sperm maturation and seminal milieu	Putative	Mechanistic tissue study	Supports inclusion of adiponectin-axis work in future sheep semen studies	(37)
Bull	Dietary vitamin D / 25-OH-D3	Whole animal	Motility; antioxidant and mineral-endocrine milieu	Improved fresh and cryopreserved semen traits	Beneficial	Dietary intervention	Justifies vitamin D stratification in breeding bulls, especially where freezability varies	(49)
Boar	Dietary vitamin D	Whole animal	Semen quality; seasonal resilience	Improved semen quality, especially in summer conditions	Beneficial	Dietary intervention	Particularly relevant for heat-stress-prone boar studs	(50, 51)
Ram	Vitamin D supplementation/extender addition	Whole animal; extender	Motility; oxidative status; post-thaw resilience	Improved semen parameters and post-thaw motility	Beneficial	Intervention + biotech	Supports ram-specific extender optimization and donor stratification	(52, 54)
Bull	Vitamin D in extender	Cryopreservation system	Viability; apoptosis-related changes	Improved post-thaw viability in some semen phenotypes	Phenotype-dependent	Direct biotech intervention	Suggests vitamin D should be tested in phenotype-stratified freezing systems	(53)

### 3.1 Cattle and bulls

In breeding bulls, IR is rarely measured directly because insulin-glucose assessments are not routinely included in breeding soundness examinations (19). Metabolic risk is therefore usually inferred from indicators such as overnutrition, excessive body fat, systemic inflammation, and diet-related metabolic disturbance (20). However, these features cannot clearly distinguish insulin dysregulation from the independent effects of adiposity, inflammation, or altered nutrient metabolism.

Thus, a useful metabolic marker should predict semen performance after processing rather than merely describe the physical condition of the donor. High-energy feeding and over-conditioning have been associated with impaired semen quality and reduced reproductive performance in cattle. A recent synthesis also suggested that paternal high-energy diets and excessive fat accumulation may affect sperm function and possibly embryo development (21), supporting the view that the metabolic condition of the bull can influence reproductive outcomes. Emerging findings in young beef bulls further indicate that overnutrition may produce measurable metabolic changes, systemic inflammation, including increased haptoglobin, and altered semen quality (20). This pattern suggests a broader endocrine-metabolic effect rather than a consequence of scrotal insulation alone.

Direct biotechnology studies provide additional support for the involvement of insulin-related signaling in cattle and bulls. Insulin has been added to semen extenders to modify sperm energy metabolism and improve post-thaw function. In an endangered bovine model, an insulin-containing extender preserved post-thaw sperm motility and plasma membrane function and improved mitochondrial membrane potential (22). However, higher insulin concentrations impaired acrosome integrity, indicating that the response is dose-dependent and that insulin should not be added empirically. Another bovine study evaluating insulin, alone or together with IGF-I, also showed that insulin-related signaling can influence post-cryopreservation sperm outcomes (23). These findings support carefully controlled, dose-specific testing of insulin in bull semen extenders.

### 3.2 Stallions and other equids

In contrast to the clear metabolic patterns observed in cattle, evidence linking insulin pathways to reproductive outcomes in stallions is less consistent. A breed-specific field study highlighted a distinct disconnect between physical adiposity and semen quality. When evaluating stallions with a normal response to an oral sugar test (OST), increased body fat or obesity was not associated with any degradation of post-thaw semen parameters. This demonstrates that, unlike in certain ruminant models where over conditioning causes local scrotal insulation and secondary germ cell damage, obesity alone in stallions does not serve as a reliable proxy for systemic insulin dysregulation or direct semen-processing risk. Consequently, physical body condition scaling cannot replace dynamic metabolic testing when forecasting an equine donor's semen cryotolerance (24, 25).

### 3.3 Boars and pigs

Boar sperm is particularly sensitive to metabolic and oxidative stress during cooling and cryopreservation (59). In this species, the composition of the processing medium appears to be an important and controllable factor. Reducing glucose concentration during pre-treatment and cooling improved post-thaw sperm quality, including motility, and was proposed to improve the fertilizing potential of frozen semen (59).

These findings suggest that excessive glucose availability during processing may increase glycolytic activity and contribute to downstream metabolic and oxidative stress (26, 27). Controlling substrate availability may therefore reduce cryoinjury in boar semen. This provides a practical example of how metabolic knowledge can be translated into a specific change in semen-processing protocols.

### 3.4 Evidence from experimental models

Experimental models play a critical role in reproductive biotechnology by isolating specific metabolic and endocrine disturbances from confounding environmental variables such as diet, season, housing, and management conditions.

#### 3.4.1 Synergistic effects of metabolic and endocrine dysfunction

Studies utilizing models that combine metabolic syndrome with hypogonadal hypogonadism demonstrate profound alterations in both spermatogenic structure and overall sperm quality. These findings indicate that metabolic burdens do not act in isolation. Instead, metabolic stress and endocrine dysfunction operate synergistically to compromise the male reproductive system. Even when resulting physical sperm impairment appears modest, these models are highly valuable for identifying the cellular mechanisms that dictate sperm survival during biotechnology procedures (28, 35).

#### 3.4.2 Core pathophysiological mechanisms

Across experimental models, three closely connected mechanisms repeatedly emerge as important contributors to reproductive dysfunction. Disturbed substrate metabolism can alter mitochondrial pathways, reduce cellular energy production, and impair the efficient use of metabolic substrates. At the same time, systemic metabolic inflammation may activate local inflammatory pathways within the male reproductive tract. These changes are often accompanied by increased oxidative stress, as metabolic disturbance promotes excessive generation of ROS that can exceed the capacity of cellular antioxidant defenses (28, 29, 32).

#### 3.4.3 The “two-hit” vulnerability model in semen processing

Spermatozoa are particularly susceptible to oxidative injury because their plasma membranes contain high levels of polyunsaturated fatty acids, while their cytoplasmic antioxidant capacity is very limited. As a result, even relatively small systemic metabolic disturbances may weaken membrane stability, mitochondrial function, and other cellular structures before semen processing begins (31, 32).

This pre-existing vulnerability becomes especially important during artificial breeding procedures. Common laboratory steps, including centrifugation, incubation, cooling, freezing, and thawing, can further increase oxidative stress and promote membrane remodeling. Metabolically compromised sperm may therefore experience a “two-hit” effect. The first hit arises from the underlying metabolic or endocrine disturbance that reduces baseline cellular resilience, while the second results from the mechanical, thermal, osmotic, and oxidative stress imposed during semen processing and cryopreservation. The combined effect may explain why semen from metabolically challenged donors often shows reduced cryotolerance, poorer post-thaw function, and lower viability.

### 3.5 Supportive evidence from human studies

Human studies cannot be directly transferred to veterinary species, but they provide useful mechanistic information. Components of metabolic syndrome, including central adiposity, dyslipidemia, hypertension, hyperglycemia, and insulin dysregulation, have been linked to altered steroidogenesis, changes in the seminal environment, and impaired sperm function (33, 34).

These studies also help distinguish probable biological mediators, such as oxidative stress and inflammation, from descriptive indicators such as body mass index alone. Collectively, the evidence suggests that IR-related metabolic disturbances may alter the functional resilience of sperm and influence how they respond to standardized processing procedures (35, 36).

### 3.6 Cross-species limitations and research priorities

Across species, metabolic effects are often more clearly reflected in oxidative balance, the seminal microenvironment, mitochondrial function, and resistance to processing stress than in semen volume or sperm concentration alone. However, many available studies are cross-sectional and are affected by season, diet, heat exposure, collection frequency, and differences in laboratory protocols. It therefore remains difficult to determine whether insulin dysregulation, altered lipid metabolism, inflammation, or another metabolic factor is primarily responsible for reduced sperm performance (21, 24).

Evidence directly linking formally measured IR to sex-sorting efficiency or sperm-selection bias remains limited in domestic animals. Nevertheless, the biological rationale is strong because sorting and selection methods enrich sperm according to motility, physical properties, and survival, all of which depend on metabolic programming and mitochondrial function.

A major research gap is the lack of studies that measure insulin-glucose status, sperm functional characteristics, and biotechnology outcomes in the same animals under standardized conditions. Addressing this gap would allow semen-processing protocols to move from average, population-based optimization toward donor-specific strategies. Metabolic phenotype could then help guide extender composition, substrate concentration, cryopreservation procedures, and quality-control testing (36). Priority should be given to species in which variation between donors in semen freezability is already a major practical concern.

## 4 Adipokines as functional signals and biomarker candidates in animal semen technology

### 4.1 Adipokine biology across species: conserved vs. divergent

Adipokines are increasingly distinguished as endocrine harmonizers connecting energy balance, inflammatory tone, and reproductive function, although their biology is not identically conserved across domestic species. Two largely conserved traits include: first, adipokines act via cognate receptors expressed in the male reproductive tract; and second, their actions extend beyond central neuroendocrine regulation to local paracrine control of steroidogenesis, sperm maturation, and sperm function. In pigs, both leptin and its receptor have been reported in ejaculated sperm, reinforcing a direct signaling interface at the gamete level rather than a solely upstream hypothalamic effect (11, 12). In rams, adiponectin receptor 1 (ADIPOR1) has been localized across multiple reproductive tissues, with detectable transcripts in testis and accessory glands, supporting that the adiponectin axis is positioned to modulate the luminal environment confronted by sperm during transit and ejaculation (37).

However, the directionality and magnitude of adipokine-sperm associations differ by species, physiological state, and sampling compartment (blood vs. seminal plasma vs. sperm-associated). Avian reproduction offers an instructive contrast, *i.e*. chemerin has been shown to hinder testosterone production in testis explants and lower sperm motility, with seminal chemerin negatively associated with sperm motility and concentration in roosters and related to lowered fertility following artificial insemination, an example where an adipokine serves as a net negative fertility signal rather than a compensatory regulator (38). Such digression appears to indicate species-specific reproductive strategies (e.g., ejaculate composition, sperm storage, and endocrine milieu), but it also emphasizes a realistic message for semen technology that “adipokine effects” should not be supposed directionally conserved across mammals and birds.

From a biotechnological point of view, conservation should be defined operationally, by whether adipokine signaling shifts measurable sperm functional modules (sperm motility patterning, membrane stability, oxidative vulnerability) in a reproducible way under optimized processing states. Recent data reinforce receptor presence and biologic plausibility in multiple species (11, 12, 37). However, robust cross-species correlation will need synchronized phenotyping (diet/season/age control) and validated assays capable of distinguishing biologically active isoforms (e.g., multimeric adiponectin) from immunoreactive but functionally heterogeneous pools.

### 4.2 Adipokines in seminal plasma

Seminal plasma is a biologically dynamic fluid rather than a passive carrier fluid. It shapes sperm survival, timing of capacitation, and resilience to *ex vivo* manipulation. Adipokines identified in seminal plasma thus require elucidation as potential ‘state reporters' of the male reproductive tract rather than only spillover from circulation. In Murrah buffalo, seminal leptin has been quantified and correlated with semen-production capacity. Higher seminal leptin was noted in bulls with reduced sperm concentration and was positively associated with sperm alterations and malondialdehyde, indicating a connection to altered spermatogenesis and enhanced lipid peroxidation rather than to increased motility kinetics (39). This pattern is mechanistically coherent: leptin can index adiposity-related inflammation and oxidative tone, however, its interpretation depends on biological compartmentalization. A correlation seen in seminal plasma may reveal testicular output, epididymal maturation conditions, or accessory gland contributions rather than sperm-intrinsic leptin signaling.

Evidence that adipokines can also serve at the sperm level reinforces the case for functional significance. In pigs, leptin receptor localization in ejaculated sperm aids the existence of direct adipokine responsiveness, potentially modulating signaling pathways related to sperm motility and acrosome function (11, 12). This matters because semen processing often selects sperm subpopulations based on motility and membrane properties; if adipokine exposure (*in vivo* or during handling) alters these traits, selection outcomes can be systematically biased.

A key procedural ramification is that seminal adipokine measurements must be interpreted alongside oxidative and inflammatory indices. The buffalo evidence is explanatory: leptin tracked with malondialdehyde and sperm abnormalities, but not with CASA sperm motility variables or seminal antioxidant enzyme activity, indicating that adipokines may map more strongly onto spermatogenic competence and oxidative insult than onto instantaneous sperm motility metrics (39). Thus, adipokines may function best as “upstream risk markers” (predicting vulnerability to process stress) rather than as direct proxies for post-processing motility. Finally, seminal plasma adipokines likely interact with other luminal factors, lipoproteins, cytokines, and extracellular vesicles, creating a complex milieu in which adipokines can shape sperm via receptor-mediated signaling or by indirectly modulating the redox-lipid microenvironment. This complexity argues for paired sampling (blood + seminal plasma) and for reporting semen collection context (season, collection frequency, diet), as adipokine concentrations may otherwise reflect confounding rather than mechanistic causality.

### 4.3 Adipokines and biotechnological performance

Adipokines interface with semen technology through two non-exclusive mechanisms: one, they report intrinsic sperm “processability” (freezability, stress tolerance), and they directly modulate sperm function when present during extender exposure, cooling, or storage. The latter is particularly relevant because extenders constitute controlled biochemical environments in which adipokine effects can be isolated. In water buffalo, *in vitro* leptin addition to extender preserved motility and viability in equilibrated (cooled) semen at low dose (10 ng/mL), whereas higher concentrations reduced motility and viability; importantly, leptin did not measurably alter DNA damage or membrane integrity across tested doses, suggesting that its main detectable impact in this context was on motility/viability dynamics during cooling rather than on chromatin stability (40). The dose-dependent trend is functionally relevant: adipokines may exhibit biphasic effects, useful within a narrow physiological window but harmful when exceeding signaling thresholds, mirroring wider endocrine biology.

Observational data on seminal plasma support performance-relevance, albeit with a different interpretation. In buffalo bulls, elevated seminal leptin has been associated with lower sperm concentration and increased alterations and lipid peroxidation (malondialdehyde), which would be anticipated to deteriorate cryosurvival and post-thaw longevity, even if baseline sperm motility seems acceptable (39). In practice, these animals may produce semen doses that pass conventional sperm motility screens yet flounder after freezing, transport, or time-dependent storage.

Other than leptin, chemerin offers a convincing example of an adipokine showing direct adverse impacts on sperm function and fertility endpoints in an agriculturally important category - poultry. Recombinant chemerin lowered rooster sperm motility *in vitro*, and chemerin-treated sperm used for artificial insemination generated reduced fertility efficacy for several days post-insemination. Also, seminal chemerin was negatively associated with sperm motility and concentration *in vivo* (38). This combines all levels, molecular signaling, semen parameters, and fertility outcomes, making chemerin a high-priority candidate for translation into semen-quality risk stratification in avian breeding systems. Therefore, such reports support a practical biotechnological hypothesis, *i.e*. adipokines may be considered as “process modifiers” able to shift the outcome distribution of cooling, freezing, and insemination, rather than simply as correlational biomarkers. Standardization is the next procedural step, *i.e*. testing adipokines under defined extender compositions, controlled cooling/freezing curves, and consistent post-thaw assessment panels to outline whether adipokines modify membrane phase behavior, oxidative flux, or capacitation timing in ways that illustrate batch losses. This is specifically relevant to veterinary semen centers, where batch failure has direct economic impacts and where a validated predictive seminal biomarker panel could have practical benefits.

### 4.4 Candidate biomarker panels for AI-studs/cryobank intake/donor stratification

A biomarker panel for semen technology should be evaluated on operational utility, predicting processing outcomes (e.g., post-thaw motility persistence), fertility endpoints (e.g., conception or hatchability), or both, with acceptable assay feasibility and reproducibility. Adipokines are attractive because they sit upstream of many sperm vulnerabilities, lipid peroxidation, inflammatory milieu, and mitochondrial inefficiency, yet a single adipokine is unlikely to provide sufficient specificity. A tiered panel approach is therefore rational.

#### 4.4.1 Tier A (core adipokines and oxidative context)

Seminal plasma leptin, alongside a lipid peroxidation marker (e.g., malondialdehyde) can flag bulls at risk of impaired spermatogenesis and oxidative insults. In buffalo, leptin tracked with abnormalities and malondialdehyde and differentiated semen-production capacity groups, supporting use in donor screening and cryobank intake decisions (39). This pairing is biologically coherent because leptin alone may indicate adiposity/inflammation, whereas malondialdehyde anchors the measurement to sperm-relevant oxidative damage.

#### 4.4.2 Tier B (receptor-informed functional markers)

Adding evidence-supported receptor axes (e.g., leptin receptor presence in sperm; adiponectin receptor presence in the tract) strengthens mechanistic explainability and facilitates donor grading by “signal responsiveness”. In pigs, sperm-associated leptin receptor evidence offers a basis for navigating semen leptin/LEPR readouts as predictors of sperm motility robustness under processing and selection (11, 12). In rams, detection of ADIPOR1 across reproductive tissues indicates that adiponectin-axis profiling could capture tract-level modulation relevant to seminal plasma composition and sperm maturation, even if sperm-level readouts remain to be validated under standardized conditions (37).

#### 4.4.3 Tier C (species-specific high impact candidates)

Chemerin attracts attention in poultry because it has been related to sperm motility impairment and lowered fertility after insemination, providing a direct translational path into donor ranking and semen-dose prediction, where rapid turnover and storage constraints are crucial (38).

Across tiers, panel design should explicitly manage confounding, as factors such as seasonality, diet, age, collection frequency, and thermal stress can each modulate adipokine profiles and semen characteristics. Thus, panels should be applied with standardized sampling (paired blood/seminal plasma where feasible), and biomarker thresholds should be optimized to local population baselines instead of being imported across breeds or management systems. The principal aim is not biomarker maximalism but decision reliability, i.e. identifying donors likely to yield stable post-processing semen doses and prioritizing interventions (dietary correction, process parameter tuning) before costly cryo- and sorting workflows are executed.

## 5 Vitamin D axis: calcium signaling, motility, and cryo/selection compatibility

### 5.1 Vitamin D *Signaling* components relevant to sperm physiology

Vitamin D biology in male reproduction is increasingly viewed as a calcium-signaling problem rather than merely an endocrine or nutritional link. The mechanistic plausibility relies on three pillars: firstly, expression of vitamin D receptor (VDR) and vitamin D-metabolizing enzymes within the male reproductive tract, facilitating local activation/inactivation; secondly, prompt, non-genomic signaling in mature sperm; and, lastly, confluence on flagellar Ca^2+^ handling, the major driver of sperm motility patterns and timing of capacitation (7, 41, 42). In human sperm, 1,25(OH)_2_D_3_ or calcitriol has been exhibited to induce a rapid increment in intracellular Ca^2+^ and to increase sperm motility, with evidence consistent with a VDR-mediated non-genomic pathway (7, 42). Such findings are functionally significant because sperm biotechnology frequently delinks sperm from their native tract environment and imposes media-dependent Ca^2+^ constraints that can shift the balance between physiological signaling and premature activation.

In sperm, entry of Ca^2+^ and homeostasis are synchronized by specialized channels and pumps, with CatSper broadly considered as a key Ca^2+^ gate for hyperactivated sperm motility and mammalian fertility. However, disruption of CatSper-mediated signaling hinders hyperactivation and fertilization competence (43, 44). Parallel systems (e.g., PMCA4 and other Ca^2+^ regulators) serve as supplementary control points that influence sperm motility and capacitation and may be sensitive to alterations in redox state and membrane composition (45). Within this architecture, vitamin D can be visualized as a context-dependent Ca^2+^ modulator, potentially beneficial where Ca^2+^ signaling is suboptimal but plausibly destabilizing where Ca^2+^ influx or capacitation tendency is already high. This bidirectionality helps interpret why vitamin D associations with semen parameters can appear consistent at a population level while intervention trials yield heterogeneous results (14, 46).

From a biotechnological perspective, the most important repercussion is that vitamin D axis effects are not likely to provide consistent increases in ‘total sperm motility'. On the contrary, they may appear as shifts in sperm motility architecture (e.g., propensity of hyperactivation), timing of capacitation, and Ca^2+^-related stress susceptibility, which are exactly the phenotypes that govern performance during cooling, freezing-thawing, and selection/sorting workflows (43, 45).

### 5.2 Cross-species evidence: domestic animals and model evidence

Cross-species data support a vitamin D-sperm association, but the evidence base remains unbalanced: most powerful for mechanistic plausibility and selected intervention studies, weaker for standardized, fertility-endpoint-anchored field validation. In mammals, foundational model evidence suggests that vitamin D signaling is biologically relevant for male fertility; VDR-null or vitamin D-deficient phenotypes have been linked with lowered fertility and abnormal sperm parameters, prompting translational exploration in domestic species (41, 47). In rams, reproductive-tract expression of VDR and vitamin D metabolizing enzymes has been studied, including detection in sperm, suggesting that vitamin D sensitivity is not confined to humans (48). More importantly, in animal production systems, dietary or systemic vitamin D interventions have begun to show effects on semen quality. A controlled study in bulls reported that dietary 25-hydroxyvitamin D3 improved semen characteristics, accompanied by increases in serum 25-hydroxyvitamin D3 and minerals, consistent with the notion that vitamin D status can shape semen parameters through mineral-endocrine coupling (49). In boars, dietary vitamin D beyond standard recommendations enhanced semen quality during summer conditions, indicating either alleviation of heat-associated reproductive stress or rectification of seasonal insufficiency effects on sperm function (50, 51).

In small ruminants, a more recent study has expanded vitamin D considerations beyond observational correlations. Supplementation of vitamin D3 has been noted to increase semen quality in rams, with suggested involvement of receptor-mediated action and oxidative-stress reduction, although fertility endpoints and external validity across management systems require further confirmation (52). Across these species, the recurring signal is most consistent for motility-linked endpoints, a biologically coherent outcome given Ca^2+^ gating of flagellar function, while effects on concentration, morphology, and endocrine profiles appear more variable (14).

However, crucial limitations must also be highlighted: firstly, vitamin D status is intertwined with season, diet composition, housing, and sunlight exposure-each of which independently impacts semen quality and oxidative tone. Secondly, some studies identified increases in semen parameters without simultaneous mechanistic readouts (Ca^2+^ flux, CatSper responsiveness, capacitation markers), limiting causal inference. Thirdly, improvements in semen “quality” do not automatically translate into field fertility unless evaluated against conception/hatchability rates, dose economics, and semen survival during storage and transport. Therefore, cross-species evidence currently supports vitamin D as a credible stratifier and modifiable exposure, but not yet as a universally effective lever across all breeding contexts (14, 49, 52).

### 5.3 Biotech leverage points: pre-collection stratification and extender calcium handling

Vitamin D becomes practically meaningful to sperm biotechnology when it predicts or modifies process losses, especially during cryopreservation and post-processing selection. Evidence is now emerging that vitamin D can be engineered into semen processing environments. In Holstein bulls, supplementation of the freezing extender with vitamin D enhanced post-thaw viability in normozoospermic samples, whereas responses differed by baseline semen phenotype, suggesting that vitamin D effects are conditional on sperm functional reserve rather than uniformly additive (53). In rams, vitamin D addition to an egg-yolk extender improved sperm motility post-freezing-thawing, reinforcing the claim that vitamin D can shift cryosurvival distributions when delivered at the processing step rather than relying merely on systemic status (54). A parallel human cryopreservation report shows enhanced post-thaw sperm motility/viability with vitamin D supplementation, supporting a conserved ‘stress-buffering' effect, potentially via antioxidant/anti-apoptotic pathways and/or calcium-linked motility preservation (55).

Mechanistically, two leverage points are most justifiable. First, pre-collection stratification, *i.e*. vitamin D status (or proxy risk factors), can be used to identify donors likely to show altered Ca^2+^ signaling and sperm motility kinetics, which directly influence post-thaw longevity and insemination dose performance. This is not an argument that vitamin D solely governs freezability; rather, vitamin D may interfere with membrane integrity and redox balance, thereby shifting vulnerability to cryocapacitation and motility collapse after thaw (14, 45). Second, extender and media design, *i.e*. because Ca^2+^ flux is paramount to sperm activation, extender composition and post-thaw handling media can be conceptualized as Ca^2+^-regulatory environments. If vitamin D enhances intracellular Ca^2+^, then extender calcium buffering, chelation capacity, and bicarbonate-driven capacitation triggers become non-trivial determinants of whether vitamin D exposure preserves function or accelerates premature activation (7, 45).

Selection and grading techniques initiate an extra compatibility constraint. Migration-based selection technologies boost sperm with robust motility and signaling competence, which may be sensitive to Ca^2+^ gating; CatSper-mediated Ca^2+^ signaling is necessary for hyperactivation and fertilization competence in mammals, and altered CatSper function reshapes sperm motility and survival under *ex vivo* conditions (43, 44). Therefore, modulation of the vitamin D axis could alter which sperm subpopulations are preferentially recovered by selection platforms, an effect that might improve functional enrichment in some contexts but could also amplify selection-induced exhaustion if Ca^2+^ signaling becomes overly permissive. The immediate trial priority is not broad supplementation, but dose-calibrated, phenotype-stratified testing using standardized cryo curves and selection endpoints, coupled to Ca^2+^/capacitation readouts that explain why outcomes shift. In veterinary terms, the goal is to tune species-adapted protocols rather than to use universal additives.

## 6 Integrating the triad: IR-adipokines-vitamin D as a systems network

### 6.1 Network models: inflammation-redox-lipidome-calcium

The IR-adipokine-vitamin D triad is best explained as a ‘coupled control system' that congregates on four sperm-connected axes, namely inflammatory tone, redox balance, lipid remodeling, and calcium (Ca^2+^) signaling. In metabolic dysregulation, IR is typically embedded within a larger syndrome of dyslipidemia and low-grade inflammation, which has been repeatedly associated with impaired semen parameters through endocrine disruption and oxidative insult pathways (56, 57). Within this environment, adipokines act less as isolated hormones and more as ‘network nodes' that encode energy balance and immune-metabolic state. Chemerin offers a mechanistically anchored example in poultry, where seminal chemerin is inversely correlated with sperm motility and concentration, and exogenous chemerin lowers sperm motility and fertility after insemination via receptor-linked effects (38). On the other hand, leptin can exhibit dose-dependent functional effects in buffalo semen extenders, suggesting that adipokine signaling can push sperm physiology toward either resilience or dysfunction, depending on the context and the intensity of exposure (40).

Coupling of redox-lipid surfaces as a principal integrator. As the sperm membrane is rich in polyunsaturated fatty acids and has restricted cytoplasmic antioxidant buffering, OS is placed as a mechanistic bridge between systemic metabolic strain and sperm damage. Thus, moderate changes in oxidative tone can transform into membrane peroxidation and downstream functional collapse (58). In biotechnology workflows, this redox susceptibility poses operationally decisive challenges where cooling/freezing and post-thaw incubation further amplify oxidative and membrane remodeling stresses, contributing to reduced fertility with cryopreserved semen (5).

Ca^2+^ signaling is then superimposed on this redox-lipid substrate. Vitamin D has been shown to increase intracellular Ca^2+^ in human sperm and is associated with motility, supporting a plausible Ca^2+^-centric interface between endocrine status and sperm movement architecture (7). Ca^2+^ flux is also tightly linked to capacitation timing and hyperactivation propensity, traits that can be distorted by cryopreservation (‘cryocapacitation'), thereby converting endocrine-metabolic differences into process-dependent performance differences (5). In this network view, the triad should not be treated as three parallel predictors; rather, IR shifts the inflammatory-redox baseline, adipokines tune immune-metabolic signaling and local reproductive-tract milieu, and vitamin D modulates Ca^2+^-dependent motility/capacitation on the background of redox-lipid stability. The primary translational implication is that biotechnology outcomes are expected to vary nonlinearly when these axes co-vary, explaining why single marker approaches often underperform in predicting cryosurvival and fertility. A critical prediction of this model is that donor classification based on the combined endocrine-metabolic profile should be tested against single biomarkers to determine whether it improves prediction of post-processing resilience; failure of multi-domain models to exceed single-marker performance would argue against the practical value of the proposed systems framework.

Despite shared mechanistic themes, endocrine-metabolic effects should not be assumed to translate uniformly across species. Both baseline vulnerability and response to treatment can be affected by ejaculate composition, sperm membrane lipid architecture, seminal plasma composition, seasonality, nutritional management, and semen-handling protocols. Accordingly, metabolic phenotyping frameworks should be calibrated within species and production context rather than imported across cattle, pigs, equids, buffalo, and avian systems without validation.

### 6.2 Endotype proposal for male breeders: field usable phenotypes

In breeding programmes, operational phenotyping should prioritize replicable classification over exhaustive mechanistic depth. A provisional endotype framework can be proposed around the triad by defining donors according to: (a) metabolic strain and oxidative vulnerability, (b) adipokine-inferred inflammatory/energy-balance signaling, and (c) Ca^2+^/motility gating propensity associated with vitamin D status. Instead of supposing that body condition alone depicts metabolic risk, evidence indicates that adiposity may not anticipate semen cryosurvival consistently across contexts, confirming the requirement for biochemical stratifiers that better approximate IR-linked physiology (56, 57).

#### 6.2.1 Endotype A (metabolic-stable/processing-robust)

Typical features of endotype A include stable semen performance across collections, low oxidative injury signals, and consistent post-thaw longevity. These donors may tolerate standard cryo curves and extenders without significant losses, and selection strategies are expected to yield predictable recovery.

#### 6.2.2 Endotype B (IR-inflammatory/redox-strained)

Endotype B is characterized by semen that may pass baseline sperm motility screens yet show enhanced process losses (cooling/freezing sensitivity, shortened post-thaw lifespan). In buffalo, seminal leptin data uphold the idea that adipokine-linked conditions can track with lipid peroxidation (malondialdehyde) and sperm abnormalities, which would possibly alter downstream cryosurvival (39). This endotype is defined by a pattern rather than a single analyte, i.e. oxidative susceptibility + inflammatory/metabolic signaling disparity + reduced functional resilience under stress.

#### 6.2.3 Endotype C (Vitamin D-Ca^2+^ dysregulation/timing-sensitive)

The key operational risk of endotype C is not necessarily poor baseline semen quality but rather impaired sperm motility architecture and capacitation timing, driven by Ca^2+^ homeostasis discrepancies. The association of Vitamin D with increased intracellular Ca^2+^ in sperm supports the plausibility that vitamin D strata may shift activation kinetics, influencing post-thaw behavior and the ‘window of usability' after processing (7).

These endotypes could be prospectively evaluated within a tiered field framework as a tiered field framework, *i.e*. Tier 1 uses collection history and basic semen QC; Tier 2 adds seminal plasma oxidative markers and a limited adipokine panel (e.g., leptin±species-specific candidates such as chemerin in poultry); Tier 3 adds vitamin D status (or proxies) when motility timing issues are suspected. Importantly, endotyping is intended to guide process choices and QC intensity, not to label donors as fertile/infertile in isolation. The value lies in predicting which donors require protocol customization to avoid predictable biotechnology losses. Such endotyping is most likely to be useful in cattle, pigs, stallions, buffalo, and poultry systems where semen doses are routinely processed, stored, or sorted under standardized commercial workflows.

### 6.3 Implications for process customization

A systems endotype framework provides a basis for ‘process personalization “process personalization” across three leverage points, *i.e*. extender formulation, cryopreservation strategy, and selection/sorting choice. First, endocrine-metabolic modulation can be incorporated into extender design in a hypothesis-driven manner. Insulin supplementation of bull semen extenders has been associated with improvements in post-thaw mitochondrial membrane potential and motility, indicating that insulin-linked signaling can be engineered at the processing step; however, such approaches require dose discipline and mechanistic monitoring because benefits may not generalize across semen phenotypes (22). Similarly, leptin shows a narrow beneficial window in cooled buffalo semen at low concentration, with higher doses impairing motility/viability, an archetypal example of endocrine signals behaving as biphasic modifiers rather than linear enhancers (40). Vitamin D has also been trialed as an extender supplement in bulls with improvements in post-thaw viability under certain conditions, implying that Ca^2+^-linked modulation can influence cryosurvival, albeit likely in a phenotype-dependent manner (53).

Second, substrate management should be treated as a controllable interface between IR-linked biology and cryobiology. In boars, reducing glucose concentration during cooling improved post-thaw sperm quality, supporting the concept that metabolic substrate oversupply during pre-freeze handling can exacerbate biochemical stress and compromise membrane/acrosome integrity (59). This provides a concrete “systems” prediction, *i.e*. endotypes marked by redox strain or glycolytic imbalance may benefit disproportionately from media designs that minimize metabolic overload during cooling and thaw recovery.

Third, selection and sorting methods may need to be matched to the donor's signaling and stress profile. Customization should therefore be judged by process-sensitive outcomes rather than static semen parameters; if Ca^2+^ signaling and capacitation propensity are shifted (e.g., vitamin D-linked Ca^2+^ gating), selection may preferentially recover sperm that are already closer to activation exhaustion, potentially shortening functional lifespan after selection. This predicts that time-to-use QC and post-selection longevity assays should be emphasized in timing-sensitive endotypes. Accordingly, customization should be evaluated with process-sensitive rather than descriptive semen endpoints (5, 58).

## 7 Implications for core sperm biotechnologies

### 7.1 Semen extenders

Semen extenders should be viewed as bioengineering environments that (a) buffer pH/osmolality, (b) supply energy substrates, (c) stabilize membranes, and (d) constrain redox drift during cooling/freezing. Their performance is therefore expected to be strongly contingent on the endocrine-metabolic state of the donor, because IR, adipokine imbalance, and vitamin D-Ca^2+^ signaling can shift baseline membrane order, mitochondrial coupling, and susceptibility to lipid peroxidation before processing begins. Contemporary literature highlights that extender design must take into account anti-cold shock and antioxidant properties while maintaining sperm acrosomal and DNA integrity, not only sperm motility snapshots (60).

The historical reliance on egg yolk and milk-derived constituents underscores their membrane-shielding properties, yet the move toward chemically defined or plant-derived substitutes has been accelerated by biosecurity and standardization pressures. A widely cited analysis of the transformation from egg-yolk to soybean/lecithin systems reveals that soy lecithin-based extenders can assist bovine semen freezing, but results differ across laboratories and bulls, consistent with an interplay between extender chemistry and sperm membrane phenotype (61). This fluctuation is particularly pertinent in endocrine-metabolic heterogeneity: i.e., sperm membranes preconditioned by dyslipidemia and oxidative stress may respond differently to phospholipid emulsions, cholesterol acceptors/donors, and cryoprotectant permeability.

A mechanistically grounded strategy is to consider extenders as modular platforms: (a) membrane stabilizers (cholesterol/phospholipids), (b) osmoprotectants/cryoprotectants, (c) substrate supply tuned to species metabolism, and (d) redox-control additives (enzymatic and non-enzymatic). Literature on domestic-animal semen cryopreservation emphasize that additive effects are seldom linear and usually rely on concentration, timing, and semen phenotype (62). This corresponds with endocrine signal biology, where leptin and insulin can exhibit slim beneficial windows and potential toxicity at higher doses in extender contexts (e.g., insulin or vitamin D supplementation studies in bulls) (53). Therefore, extender comparisons should prioritize durability and functional resilience after thaw over immediate motility alone (5, 60).

### 7.2 Cryopreservation

Cryopreservation continues to be the major obstacle in semen biotechnology as it enforces successive membrane phase transformations, osmotic stress, and oxidative stress that deteriorate fertility relative to fresh semen through both loss of viability and sublethal dysfunction among survivors (5). Classic mechanistic syntheses highlight that sperm membranes are complex and that fluctuations can originate at multiple levels (dilution, cooling, cryoprotectant exposure, freezing, thawing), making cryosurvival a distributed phenotype rather than a single-point characteristic (5, 63). This distributed vulnerability is precisely where endocrine-metabolic state becomes functional, *i.e*. small baseline shifts in lipid composition, antioxidant reserve, and mitochondrial coupling can be amplified across the freeze-thaw cascade.

A principal ramification is that “freezability” should not be considered as a fixed individual feature. It is the pressing consequence of sperm structural modules interacting with a specific protocol. Contemporary literature in farm animals and in bull cryobiology particularly emphasize that molecular biomarkers and functional features can help predict cryotolerance and that protocol innovations increasingly target membrane stabilization and oxidative control (64, 65). Cholesterol-centric approaches offer a mechanistically clear example. Cholesterol stabilizes membranes at low temperature, and cholesterol-loading strategies have been put forward exclusively to improve cryosurvival and post-thaw function across species, positioning membrane cholesterol as both a vulnerability and an adjustable target (15).

The metabolic axis intersects cryopreservation through substrate handling, too. In boars, lowering glucose in pre-treatment media during cooling enhanced post-thaw sperm motility and was proposed to increase fertilization outcomes with frozen sperm, a report compatible with limiting metabolic overdrive and downstream stress during cooling transitions (59). This observation is specifically pertinent to IR-like phenotypes, where substrate flux and redox leakage may already be maladaptive; glucose moderation becomes a process-control lever rather than a nutritional footnote.

Practically, cryopreservation protocols should include stress-sensitive QC, *i.e*. post-thaw longevity, mitochondrial membrane potential, acrosome responsiveness, and oxidative indices, because endocrine-metabolic perturbations may produce normal immediate sperm motility yet poor durability and fertilizing lifespan. Conventional cryobiology should move toward phenotype-aware QC whenever immediate sperm motility fails to predict durability or fertility (64, 65).

### 7.3 Sex-sorting

Flow-cytometric sex-sorting is functionally effective because it gives high X/Y purity by measuring DNA content after Hoechst staining, yet it inflicts an unambiguous stress signature, *i.e*. dye exposure, laser probing, hydrodynamic/mechanical forces, and typically low-dose packaging followed by specialized insemination strategies (66). The crucial question for endocrine-metabolic interfacing is not whether sorting “harms sperm” in general, this is context- and protocol-dependent, but which functional modules are most vulnerable and whether metabolic phenotypes shift the tolerance threshold.

Technical reviews explain the key engineering limitations (orientation, speed, purity, and dose economics) and highlight that changes in laser power, dilution, and post-sort handling materially affect post-thaw motility and viability of sexed semen (67, 68). Schenk et al. (68) reported that manipulating dilution and laser parameters affected the percentage of progressively motile sperm after thawing, highlighting that sorting is not a single insult but a set of adjustable stressors with measurable functional costs. Mechanistic work in stallion sperm has further demonstrated increased OS after sorting and explored relationships between ROS production and DNA damage, reinforcing oxidative load as a plausible mediator of post-sort dysfunction (69).

These points align with endocrine-metabolic logic: IR and adipokine imbalance appear to co-travel with increased oxidative tone and altered mitochondrial coupling, shrinking the buffer against an already ROS-amplifying process. Thus, sex-sorting programmes may gain from pre-sort stratification using oxidative and mitochondrial markers, and from post-sort protocols that prioritize redox stabilization and rapid recovery. This is consistent with broader farm-animal cryopreservation evidence that molecular biomarkers can predict cryotolerance and with the known vulnerability of sexed semen to reduced fertility and reduced longevity in the female tract when dose and handling are suboptimal (5, 65).

A second implication concerns selection bias, *i.e*. sorting is indifferent to metabolic phenotype in principle, yet it enriches sperm that survives a high-stress pipeline. If endocrine-metabolic state shifts subpopulation structure (e.g., proportion of mitochondrially competent cells), sexed semen batches may show increased between-donor variance and may require donor-specific parameter tuning (laser/dilution/recovery and freezing curve) to reduce failure rates. In this setting, ‘one protocol fits all' is likely to underperform compared with phenotype-informed calibration.

### 7.4 Sperm-selection techniques

Sperm selection technologies enrich subpopulations based on superior motility, morphology, membrane integrity, or DNA stability. Their relevance to animal semen technology has expanded from assisted reproduction in humans to domestic and wild species, including applications that prioritize DNA integrity and functional competence (70). The endocrine-metabolic interface is immediate, *i.e*. selection algorithms are, in effect, filters over sperm metabolic performance, because motility and survival depend on ATP supply, membrane stability, and controlled Ca^2+^ signaling. Metabolic phenotypes, therefore, have the potential to distort selection outputs, either by reducing recovery (fewer robust sperm) or by skewing recovered subpopulations toward ‘fast but fragile' cells with limited post-selection longevity.

Colloid-based selection (single-layer centrifugation, SLC) illustrates a comparatively field-ready approach. Practical guidance in equine reproduction describes SLC as a method to separate sperm with better motility and functional attributes through colloids, supporting its translational value as a pre-freeze or pre-insemination enrichment step (71). In stallions and other species, SLC has been reported to improve sperm parameters by reducing dead/dying sperm and contaminants, and cattle data show that SLC can select sperm populations with improved functional markers (72). Such methods are plausibly advantageous in endocrine-metabolic risk settings, as they may reduce ROS-generating enzymes and pro-inflammatory debris that amplify oxidative drift during storage and freezing.

Microfluidics provides a different value proposition that reflects on low-shear, non-centrifugal selection that aims to enrich motile, functionally competent sperm, with growing animal-specific evidence and device testing in boars and other species (70, 73). A key systems issue is compatibility, *i.e*. device materials, channel geometry, and media composition can interact with sperm membranes and metabolic status; thus, endocrine-metabolic phenotyping can help explain why some donors show improved DNA integrity and IVF outcomes post-selection while others show modest gains or reduced recovery.

Current comparative syntheses in selection technologies caution that superiority over conventional swim-up and density gradients is not universally established across endpoints and contexts (74). This is exactly why selection experiments in endocrine-metabolic contexts should report not only recovery or motility gain, but also post-selection durability, oxidative load, and downstream fertilization performance. In animal semen technology, the tactical move is thus not to replace conventional methods indiscriminately, but to employ selection strategies as endotype-matched interventions so that oxidative-risk donors may benefit more from low-shear microfluidics or colloid enrichment prior to freezing; donors with timing-sensitive sperm capacitation propensity may require selection methods that minimize premature activation and maintain post-selection longevity (70, 71).

### 7.5 New fertility markers and prediction of animal fertility

Predicting fertility from semen remains a central unmet need because conventional semen analysis takes into account only part of fertilizing capacity of a male, and because biotechnological manipulations bring in stressors that disconnect baseline semen characteristics from post-processing performance. Methodological reviews highlight that semen analysis needs more stringent QC and extended functional assays to enhance fertility inference, including membrane integrity, acrosome status, mitochondrial potential, chromatin structure, and oxidative stress (75). In cattle, functional and molecular traits linked to fertility and cryotolerance are being actively assessed, while recognizing that several proposed markers still lack field verification and remain context-specific (76).

The endocrine-metabolic triad extends the biomarker expanse in two ways. Firstly, it encourages the identification of pre-collection phenotyping markers that predict processability (freezability, sex-sorting tolerance, post-thaw longevity) instead of fertility in isolation. Secondly, it emphasizes seminal plasma as a reporting section where metabolic-inflammatory-redox signatures may trace sperm vulnerability. Proteomics and multi-omics approaches are increasingly proposed to identify seminal plasma or sperm proteins associated with semen parameters and fertility, emphasizing the plausibility of “molecular fertility signatures” (77). At the same time, actual application in breeding systems requires graded logic: field-feasible markers for routine donor management, followed by advanced molecular profiling for high-value donors or troubleshooting.

On the analytics side, CASA remains fundamental in AI centers, yet literature highlights that CASA results are sensitive to methodological errors and that their relationship to field fertility is varied; improved interpretation needs careful standardization and multivariate modeling (78). Recent literature also stresses multivariate frameworks that combine CASA with other semen endpoints to improve fertility inference, corresponding with a systems approach rather than a single-parameter threshold model (79). Future predictive models should therefore be benchmarked against routine semen analysis using discrimination metrics relevant to field deployment, such as gain in post-thaw classification accuracy, recovery prediction, or fertility calibration.

A systems-network view suggests a proposed marker architecture for future validation (a) functional stress marker (mitochondrial potential, oxidative indices, post-thaw longevity), (b) semen-environment markers (selected seminal plasma proteins/adipokines), and (c) process metadata (freezing curve, extender type, sorting/selection exposure). This architecture is compatible with the trend toward integrating molecular biomarkers into cryotolerance prediction in farm animals (65) and addresses the operational reality that fertility prediction must be process-aware, predicting performance after chilling/freezing/sorting, not just at ejaculation.

## 8 Comparative translation and veterinary research priorities

### 8.1 Contribution of veterinary models unique to reproductive biotechnology

Human fertility trials that interrogate IR, adipokines, and vitamin D frequently stall at the interface between biological plausibility and clinical heterogeneity. The relative benefit of animal systems is not simply mechanistic advantage; it is the ability to regulate exposure intensity and timing, repeatedly sample within-individual semen phenotypes, and quantify fertility endpoints at scale under standardized handling. In humans, metabolic syndrome and obesity are often related to decline of semen parameters and sperm DNA integrity, yet the causal chain is difficult to resolve because diet composition, physical activity, sleep, medications, and comorbidities cluster non-randomly (56, 57, 80). Animal models can seclude single-node perturbations (e.g., caloric excess, insulin dysregulation, micronutrient manipulation) while keeping up environment and collection conditions unchanged, circumstances under which semen processing steps (cooling, freezing, incubation) can be applied equally across individuals.

Secondly, process-aware phenotyping is another distinctive contribution. Human ART experiments usually consider semen as the endpoint, whereas semen in clinical practice is routinely processed and subjected to oxidative and osmotic stress. Literature on cryopreservation point out that freezing-thawing enhances OS and can incite SDF and apoptosis-related signatures, indicating that baseline semen quality may be an insufficient predictor of post-processing competence (81, 82). Animal studies enable direct assessment of whether metabolic endotypes predict processing losses, a translationally pertinent phenotype for sperm banking and for clinics using cryopreserved sperm.

Thirdly, animal systems allow coupled sampling of blood-seminal plasma-sperm compartments, elucidating whether endocrine-metabolic cues act as systemic correlates or local effectors. Seminal adipokines have been suggested as functional modulators rather than passive spillover, but human data are variably confounded by abstinence interval and genital-tract inflammation (83). Controlled animal sampling can determine whether adipokine signatures track epididymal/seminal plasma environments that shape sperm resilience.

Finally, comparative designs can examine bidirectional transfer, *i.e*. animal work can characterize mechanistic endpoints (e.g., oxidative susceptibility, motility kinetics, post-thaw longevity) that human testing can adopt, while human observational signatures can guide which animal exposures and endpoints are most clinically pertinent (80, 84). The strategic value is a shared endpoint architecture that links endocrine-metabolic biology to the real-world stressors imposed by ART workflows.

In human male infertility, these mechanisms may be clinically relevant because metabolic dysfunction can alter semen quality, sperm redox balance, and DNA integrity, which may influence sperm functional competence (80, 82). Endocrine-metabolic phenotyping may therefore help identify biologically distinct subgroups of men who are more vulnerable to processing-related stress during sperm preparation, cryopreservation, or selection for IVF/ICSI (80, 82). However, these applications remain investigational and require prospective validation against clinically relevant ART outcomes, including fertilization, embryo development, pregnancy, and live birth (85).

### 8.2 Priority study designs for veterinary reproductive biotechnology

The central failure mode of earlier ‘supplement-and-measure' trials is the supposition of unvarying biology. Endocrine-metabolic modulators seldom function linearly; they work through coupled networks of redox balance, lipid remodeling, and calcium signaling, giving rise to responder and non-responder stratification. Therefore, trial design should pivot from average treatment effects to stratified, phenotype-informed conclusion. Literature on metabolic syndrome reinforce consistent relations with semen impairment, but they also emphasize on heterogeneity in endocrine outputs and semen endpoints, indicating clear cut mechanistic pathways among participants (56, 57).

#### 8.2.1 Design priority 1: endotype-stratified RCTs

Participants should be stratified by authenticated IR proxies (e.g., HOMA-IR or comparable indices), inflammatory markers, and baseline oxidative vulnerability (preferably semen-based). This is necessary because oxidative insult and mitochondrial incompetence are probable mediators connecting metabolic dysfunction to sperm impairment and to processing losses during cryopreservation (81, 82).

#### 8.2.2 Design priority 2: multi-compartment profiling

Parallel measurement of serum and seminal plasma adipokines (e.g., leptin, adiponectin) should go with semen functional modules. Literature on semen adipokines indicate concentration-dependent and context-dependent associations with sperm motility and other functions, making segmented resolution critical for mechanistic interpretation (83, 86).

#### 8.2.3 Design priority 3: process-aware primary outcomes

Instead of depending merely on traditional semen analysis, trials should consider endpoints that reveal ART-relevant stress: post-processing sperm motility persistence, mitochondrial membrane potential, OS indices, and DNA integrity pre- and post-processing (including after freeze-thaw where clinically applicable) (81, 82).

#### 8.2.4 Design priority 4: reproductive outcomes with mechanistic anchors

Vitamin D testing depicts why only semen endpoints can be deceptive. An RCT found no overall increment in semen parameters with vitamin D supplementation, while indicating likely signals in particular subgroups and reproductive outcomes that warranted further validation (7). Future trials should predefine subgroup analyses (e.g., vitamin D-deficient oligozoospermic men, IR-high endotypes) and relate clinical outcomes (pregnancy/live birth where feasible) to mechanistic intermediates (Ca^2+^-linked motility architecture, oxidative vulnerability).

Across designs, the aim is not wider supplementation but precision exposure testing, recognizing which endocrine-metabolic endotypes show measurable improvements in sperm processability and reproductive outcomes, in sperm processability and reproductive outcomes, while circumventing null trials driven by biological averaging.

### 8.3 Reverse translation back to animal reproduction

Human trial preferences should in turn cater to animal reproduction through three resultants: endpoints, stratification logic, and risk management. Firstly, the most transferable human innovations are functional semen endpoints that are reliable under processing strain. In humans, literature on cryopreservation underlines OS, antioxidant exhaustion, and the persistence of deleterious sperm dysfunction after thaw, characteristics that resemble losses noticed in livestock protocols and that can be determined without species-specific ART framework (81, 82). Applying post-thaw durability curves, oxidative markers, and DNA integrity indices as standard “stress tests” can enhance donor grading and protocol streamlining in animal AI centers, where fertility prediction remains sub-optimal (80). Secondly, human stratified-trial reasoning can be reverse-translated into donor endotyping. Human reviews highlight that obesity and metabolic syndrome impact sperm DNA integrity and semen quality, and recent syntheses highlight that intervention effects on male fertility are diversified, accurately the pattern anticipated when multiple metabolic pathways coincide (80, 84). In animals, where recurrent collection is attainable, donors can be ranked by stability of semen performance and by biochemical risk markers (oxidative vulnerability; adipokine panels adapted to species), allowing targeted protocol optimization rather than uniform extender and freezing curves. Thirdly, human trial safety concepts can inform animal interventions. Vitamin D supplementation trials illustrate that endocrine modulation may not improve semen parameters globally and that benefits may be context-dependent (7). This argues against universal additive use in extenders or diets without donor-level evidence. Instead, an evidence-to-practice pipeline is recommended, (a) identify an endotype linked to consistent processing losses, (b) test mechanistically aligned interventions (substrate moderation, membrane stabilization, redox control, calcium-handling adjustments) with functional endpoints, and (c) scale only when fertility endpoints confirm benefit. Finally, reverse translation should emphasize comparability of metadata: diet composition, seasonality, thermal load, collection frequency, and processing timelines must be reported and standardized, because these variables confound endocrine-metabolic signals and semen traits in both humans and animals (56, 57). The net effect is a shared translational framework, *i.e*. endocrine-metabolic endotyping becomes a decision tool for semen technology optimization, rather than a descriptive label that fails to improve fertility outcomes. Three limitations currently constrain field translation. First, endocrine-metabolic variables are often inferred from imperfect proxies rather than measured directly in breeding males. Second, many studies report semen quality endpoints without matching them to processing-specific or fertility outcomes. Third, assay heterogeneity across adipokines, oxidative markers, CASA settings, and post-thaw readouts limits threshold portability across breeds, species, and laboratories. These constraints should be recognized not as peripheral caveats, but as the principal reasons why biologically plausible signals have not yet matured into standardized biotechnology decision tools.

## 9 Methodological standards and reporting checklist

### 9.1 Minimum dataset for cross-study comparability

Cross-study synthesis is routinely undermined by incomplete metadata, particularly when endocrine-metabolic exposures are being linked to sperm processing outcomes. At minimum, reports should standardize and disclose, *i.e*. species/breed, age, body condition/adiposity proxy, health status/medications, housing and thermal load, diet composition and supplementation, season/photoperiod, ejaculation frequency and abstinence interval, collection method, time-to-processing, dilution ratio, extender identity (full composition where possible), storage temperature profile, and operator/instrument identifiers. Such “process metadata” is essential because semen evaluation is highly method-dependent and requires strict quality control to interpret fertility relevance.

### 9.2 Core sperm endpoints: adopt a function-rich set

Conventional parameters (volume, concentration, total/ progressive motility, morphology) should be treated as baseline descriptors rather than sufficient surrogates of fertilizing competence. A function-rich core panel is recommended, *i.e*. plasma membrane integrity, acrosome integrity/reactivity, mitochondrial membrane potential, OS indices, and (where feasible) chromatin/DNA integrity, reported both at baseline and after relevant processing steps (cooling, freeze-thaw, sorting/selection). Contemporary methodological reviews emphasize that expanded assay panels are necessary because “normal” semen traits can coexist with sublethal dysfunction that only becomes evident under processing stress.

### 9.3 Fertility endpoint hierarchy: align to the biological question

Endpoints should be pre-specified in a hierarchy that matches use-case, *i.e*. (Tier 1) laboratory/processing outcomes (post-thaw longevity curves, recovery yield after sorting/selection); (Tier 2) intermediate functional endpoints (capacitation competence, oocyte penetration or IVF metrics where used); (Tier 3) field fertility outcomes (non-return rate, conception rate, litter size/hatchability). Cryopreserved semen is known to lose fertility via both reduced viability and sublethal dysfunction among survivors; therefore, field outcomes should not be inferred from motility alone.

### 9.4 Bias control: confounding and analytical transparency

CASA outputs are highly sensitive to chamber type, frame rate, optics, temperature, and analysis settings; thus, instrument configuration, thresholds, and QC procedures must be reported explicitly, and raw video storage is preferable when feasible. For cryobiology, freezing/thawing curves (rates, holds), cryoprotectant concentrations, equilibration times, and ROS-management steps should be transparently documented because omics and functional signatures of cryo-tolerance are protocol-dependent.

## 10 Future directions: metabolic personalization to sperm biotechnology

“Metabolic personalization” recasts sperm processing as a donor-by-protocol associated issue, where endocrine-metabolic setting is considered as a manageable source of variance rather than nuisance noise. Further methodological step is to proceed from static semen descriptors toward stress-response phenotyping, *i.e*. brief, standardized challenge assays (cooling, controlled oxidative load, freeze-thaw micro-tests) coupled with functional endpoints (mitochondrial membrane potential, membrane/acrosome integrity, oxidative indices, and post-thaw longevity curves) that better capture harmful dysfunction, an acknowledged operator of lowered fertility with cryopreserved semen.

Mechanistically oriented protocol customization can then be followed along three axes: i) membrane engineering: cholesterol regulation (e.g., cholesterol-rich cyclodextrins) stands a controllable lever for enhancing thermo-resistance in spermatozoa with low intrinsic cholesterol:phospholipid ratios. However, it should be positioned with donor grouping to avoid concealing capacitation competence; ii) redox management: during cryopreservation, OS is progressively defined at molecular level, facilitating the creation of extenders and thaw recovery media that restrict ROS build-up without driving reductive remnants; iii) selection/sorting compatibility:, low-stress selection platforms (including microfluidics) should be assessed as endotype-specific tools, handy for donors whose semen is disproportionately destabilized by centrifugation or oxidative shift.

Functionally, this schedule needs standardized reporting of processing loops and assay settings, as well as predictive modeling that combines metabolic surrogates with semen functional frameworks for prognosis of process-normalized fertility.

## 11 Conclusions

In sperm biology, endocrine-metabolic modulation should be considered as a design variable rather than an external confounder. IR-associated metabolic strain is frequently aligned with oxidative instability and changed energy handling, initiating a “pre-loaded” vulnerability that becomes operationally visible when sperm are cooled, frozen-thawed, sorted, or selected. Lowered fertility after cryopreservation is now well thought out as the joint consequence of post-thaw cell loss and sublethal dysfunction in the surviving fraction, reiterating the necessity to phenotype resilience rather than to solely rely on motility profile (5). A systems explanation of the IR-adipokine-vitamin D triad projects non-linear interplays across redox balance, membrane stability, and Ca^2+^-gated motility/capacitation. For veterinary reproductive biotechnology, the pivotal follow up action is prospective testing of whether endocrine-metabolic endotyping enhances donor grading, post-processing resilience, and fertility prediction beyond mere traditional semen evaluation. In species-specific semen handling workflows, if this added value is exhibited, then metabolic phenotyping could develop into a useful decision-support approach in animal reproduction if its incremental value is confirmed prospectively.
